# Comparison of glucose concentrations in samples frozen for varying durations and subjected to freeze-thaw cycles using 2 assay systems

**DOI:** 10.3168/jdsc.2024-0729

**Published:** 2025-02-20

**Authors:** Anay D. Ravelo, Megan Ruch, Isaac J. Salfer, Luciano S. Caixeta

**Affiliations:** 1 Department of Veterinary Population Medicine, University of Minnesota, Saint Paul, MN 55108; 2 Department of Animal Science, University of Minnesota, Saint Paul, MN 55108

## Abstract

Although laboratory best practices recommend that total storage time and number of freeze-thaw cycles should be minimized before analysis of metabolites within plasma, the specific impacts of storage time and freeze-thaw cycles on apparent glucose concentrations in bovine plasma have not been well quantified. Additionally, within the field of dairy science, multiple assay systems for glucose concentration analysis are used, yet these assays have not been directly compared with each other. Thus, the first objective was to investigate the stability of glucose in samples that have been frozen for an increased duration or frozen and thawed up to 4 times. The second objective was to investigate the agreement between 2 assay systems for glucose measurement: the peroxidase and glucose oxidase (PGO) assay system and the hexokinase reaction (HK) assay system. Blood samples were collected into sodium fluoride (NaF) tubes from 166 dairy cows, across different lactation stages, on 3 farms over a 2-d interval. After centrifugation, plasma was separated into 4 aliquots. One aliquot was used to determine the impacts of up to 4 freeze-thaw cycles, and the first measurement from this aliquot along with the other 3 aliquots were used to investigate potential glucose degradation in aliquots frozen for different lengths of time, including 2, 3, 4, and 16 wk. All samples were analyzed using both the PGO and HK assay systems. Bland–Altman plots, Pearson correlations, and paired *t*-tests were used to compare the effects of storage time and freeze-thaw cycles. Additionally, a linear mixed model with the fixed effect of either storage time or freeze-thaw cycle and the random effect of cow within farm was used to consider the effect of each of these factors on measured glucose concentrations. For comparisons between assay systems, a linear mixed model was used with the fixed effect of assay system, time, frozen or freeze-thaw cycles depending on the model, their interaction, and cow within farm as a random effect. Storage time and freeze-thaw cycles influenced glucose concentration measured by the PGO and HK. Concentrations of glucose both increased and decreased with increasing storage time and freeze-thaw cycles and compared with the initial quantification. Apparent glucose concentration was more variable across all aliquots when the HK assay (± 0.14 mmol/L) was used compared with the PGO assay (± 0.06 mmol/L). The HK assay continuously yielded lower concentrations of glucose compared with the PGO assay. Overall, glucose concentrations can be variable over different storage times and freeze-thaw cycles; however, they cannot be compared across the PGO and HK assay systems.

## Highlights

• Glucose concentrations were minimally affected by length of storage time.
• Glucose concentration was minimally affected by freezing and thawing.
• Assay systems for glucose quantification did not have good agreement.

## Graphical Abstract

**Figure:**
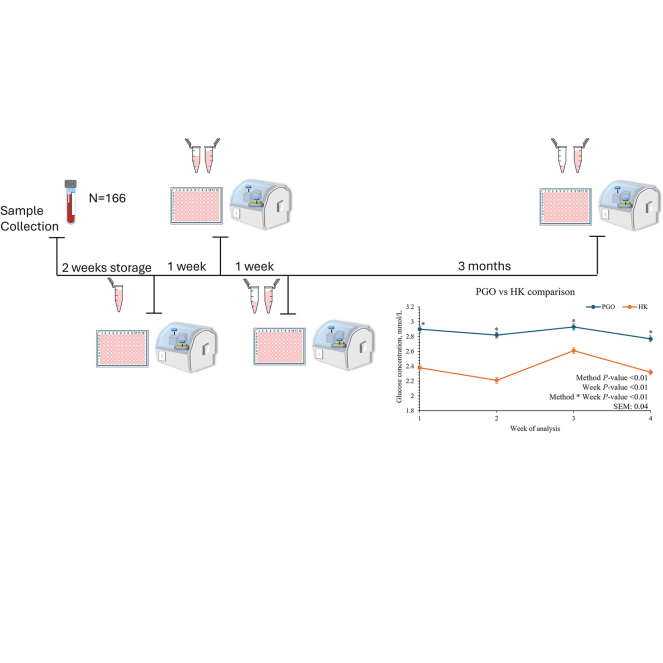
**Summary:** Blood samples were collected from 166 cows into sodium fluoride tubes. One aliquot was used to compare glucose concentrations between repeated freeze-thaw cycles (1-4), and 3 were used for comparison of storage time (2, 3, 4, or 16 weeks) at -20°C. Two assay systems and reactions were used for quantification of the glucose concentration in the aliquots. It was observed that freeze-thaw cycles and storage time had minimal effects on glucose concentration measured when using the peroxidase and glucose oxidase enzyme (PGO). Concentrations measured over time for storage and freeze-thaw cycles using the hexokinase assay system (HK) were more variable and lower compared with PGO.

Study protocols in dairy cows often require plasma samples to be frozen immediately after collection for further analysis (Abou-Rjeileh et al., 2023; Arshad et al., 2023). Freezing and storing samples allows them to be analyzed in a shorter window of time, reducing potential assay variation. However, in long-term studies, freezing time differs between samples collected on different days of the experiment, with samples collected and stored at the beginning of a trial usually having a longer storage time than samples collected toward the end of the trial. Although impacts associated with storage time could be partially reduced by including a period effect in some study designs, they could still add to residual error and potentially lead to increased variation in longitudinal studies. Additionally, during the laboratory analysis, some samples may need to be thawed numerous times to be reanalyzed due to inconsistent laboratory techniques and unusual results or because several analyses need to be conducted using the same sample aliquots. Even though protocols typically advise against freeze-thaw cycles to maintain sample integrity, few studies have considered their effects on glucose quantification (Haid et al., 2018; Buchanan et al., 2022).

Across laboratories, a variety of different assay systems of analyzing glucose concentrations can be used depending on the resources available. Two popular assays used for glucose quantification involve using a peroxidase and glucose oxidase (**PGO**) enzyme preparation or a hexokinase reagent (Fiedorova et al., 2022). Both assays use colorimetric assays and have been widely used in dairy cow research (Mann et al., 2016; Martin et al., 2021). Although these are popular assay systems, to our knowledge, no research has validated whether the concentrations of glucose quantified by each assay are comparable.

Therefore, the objectives of this study were to determine the impacts of storage time, number of freeze-thaw cycles, and the type of enzyme-based assay system on the apparent glucose concentration in blood collected from dairy cattle. It was hypothesized that increased storage time and a greater number of freeze-thaw cycles would both linearly decrease apparent glucose concentrations. It was also hypothesized that apparent glucose concentration would not differ based on enzyme-based assay systems.

Sample size was calculated based on previous studies investigating the use of a glucose meter to measure glucose concentrations in cows compared with a reference assay. These studies observed a difference of 0.26 mmol/L between 2 assay systems of glucose measurement (Mair et al., 2016) and an SD of 1.10 mmol/L (Wittrock et al., 2013). Based on these results, we calculated an effect size of 0.24 for Cohen's d, obtained by dividing 0.26 mmol/L by 1.10 mmol/L. Thus, when using a paired *t*-test it was calculated that 146 samples would be necessary to detect the expected difference in concentration of glucose with a power of 0.80 and an α of 0.05 based on these assumptions. To account for potential hemolysis and blood clots in samples, an additional 20 samples were collected to help make sure there were enough samples.

The samples were collected from cows already enrolled in 2 ongoing observational studies within our research group. The University of Minnesota Institutional Animal Care and Use Committee approved procedures for animal care and handling in these studies (protocol numbers 2211-40523A and 2303-40919A). Blood was sampled from dairy cows (n = 166) from a coccygeal vessel into sodium fluoride tubes with potassium oxalate (BD Vacutainer Fluoride Tubes, Becton Dickinson; Bayot and Tadi, 2023) on 3 different farms (farm A: n = 129; farm B: n = 24; farm C: n = 13) within a 72-h timeframe. In farm A, samples were collected from both dry (n = 41; days carrying calf = 261 ± 29; 48.8% were multiparous) and fresh cows (n = 88; 6 ± 3 DIM; 59.0% were multiparous). In farm B, samples were collected from early-lactation cows (n = 24; 50 ± 16 DIM; 87.5% were multiparous). In farm C, samples were collected from both dry (n = 8; days carrying calf = 272 ± 10; 75.0% were multiparous) and fresh cows (n = 5; 14 ± 2 DIM; 60.6% were multiparous). Samples were stored on ice and transported to the laboratory within 3 h of collection, where they were centrifuged at 2,000 × *g* for 15 min at 4°C. The plasma was separated into 4 aliquots (1 mL each) and stored in polypropylene microcentrifuge tubes (111562LK, Globe Scientific. Inc.) at −20°C until analysis.

To determine the influence of freeze-thaw frequency on glucose concentration, one aliquot of plasma was thawed for the first time at room temperature on d 14 and 16 after sample collection, and glucose concentration was measured using PGO enzyme and hexokinase assay systems (**HK**; freeze-thaw cycle 1 [**FTC1**]). Samples were refrozen 3 h after thawing, and then re-thawed at room temperature 1 wk later for a second measurement of glucose concentration (freeze-thaw cycle 2 [**FTC2**]). These steps were repeated once more 1 wk later (freeze-thaw cycle 3 [**FTC3**]) and again 16 wk after initial sample collection (freeze-thaw cycle 4 [**FTC4**]). To consider the possible influence of storage time on glucose concentration without the influence of the freeze-thaw cycles, a fresh sample was thawed for the first time at each analysis time point (14 to 16 d after collection [**ST2**], 3 wk [**ST3**], 4 wk [**ST4**], and 16 wk [**ST16**] after the day of original freezing).

All samples were analyzed in tandem using 2 different assay systems to determine the influence of the assay system on apparent glucose concentrations. The assay systems included a colorimetric microplate reader (Eon, BioTek Instruments) assay using a PGO enzyme preparation (Sigma Aldrich, St. Louis, MO; Shaker and Swift, 2023), and a small-scale chemistry analyzer (Chemwell-T Chemistry Analyzer, Awareness Technology Inc.) using hexokinase reagent (C-124-07, Catachem Inc., Fiedorova et al., 2022). Briefly, for the microplate analysis, 5 µL of standards and samples were loaded onto a 96-well plate (Greiner Bio-One, Frickenhausen, Germany). Then, 250 µL of PGO reagent was added using a multichannel pipette, and the plate was shaken for 10 s using the mix feature in the colorimetric microplate reader (Eon, BioTek Instruments). The plate was allowed to sit for 45 min in the dark, and absorbance was read at a wavelength of 450 nm. The chemistry analyzer (Chemwell-T Chemistry Analyzer, Awareness Technology Inc.) was calibrated using the manufacturer instructions. The analyzer was loaded with samples and reagent. With automated pipetting, the analyzer loaded 300 µL of hexokinase reagent into cuvettes (CW-T Cuvettes, United Scientific). The absorbance of each well with reagent was read at 350 nm, 10 µL of sample was added to the corresponding well, and the mixture was agitated. The mixture was allowed to sit for 5 min before the final absorbance was read at the same wavelength.

A standard curve with prepared glucose concentrations of 0, 1.1, 2.2, 3.3, 4.4, and 5.5 mmol/L was used for determination of glucose concentrations within the PGO assay. The standards for the HK reagent had 4.38 to 6.32 mmol/L for the lowest standard and 14.76 to 18.54 mmol/L for the highest standard. Because the standard curve for the HK analysis was a 2-point curve that did not encompass the expected range for glucose concentration in cow plasma, the standards that were prepared for the PGO analysis were also analyzed using the HK assay and absorbances were used to create a standard curve for the HK assay. When analyzed through the small-scale chemistry analyzer, the standards demonstrated high accuracy (correlation coefficient of 0.99) and low variation (CV of 5.3%). The CV for the standards with the PGO assay was 4.98%, and the CV for the HK assay was 5.3%.

Two pool samples consisting of 5 randomly chosen samples with low visual evidence of hemolysis were analyzed for every analysis time point (storage time or freeze-thaw cycle). The pool samples had intra- and interassay CV ranging from 1.09% to 4.58% and 1.90% to 7.66%, respectively, when using the PGO procedure. They had intra- and interassay CV ranging from 0.85% to 1.89% and 1.50% to 7.46%, respectively, when using the HK procedure.

All statistical analysis were performed using R studio (v 4.4.1, https://www.r-project.org/). Bland–Altman (**BA**) plot, matched *t*-tests, and Pearson correlations, were used to compare glucose concentrations of samples stored for 2 wk compared with the other storage times and the first freeze-thaw cycle to those from repeated freeze-thaw cycles. The BA plots were created using the *ggplot*() function from the *ggplot2* package (Wickham, 2016) and the Pearson correlations were illustrated using the *ggscatter*() function from the *ggpubr* package (Kassambara, 2016). Additionally, linear, quadratic, and cubic contrasts were analyzed using a mixed model with the *lmer*() function of the *lme4* package (Bates et al., 2015). Each model included the fixed effect of either storage time or freeze-thaw cycle and the random effect of cow within farm to compare concentrations at the different times of analysis. For comparisons of the PGO and HK assay systems, the mixed linear model included the fixed effect of assay system, time of analysis, and their interaction, as well as the random effect of cow within farm. A BA plot was also created for the comparison between assays. For all BA plots and mixed models, samples with studentized residuals exceeding ± 3 SD from the mean were removed, and a Tukey adjustment was applied for pairwise comparisons in mixed models.

Glucose concentrations quantified with PGO at ST3, ST4, and ST16 were less than the concentrations measured for ST2 (Table 1; Figure 1). For samples analyzed with HK, the difference between ST2 compared with ST3, ST4, and ST16 were 0.16 (*P* < 0.01), −0.18 (*P* < 0.01), and 0.08 mmol/L (*P* < 0.01), respectively (Table 1; Figure 1). The effect of storage time was significant (*P* < 0.01) in both assays. Although, the apparent glucose concentrations measured with PGO decreased with the increase in storage time, concentrations measured with HK both increased and decreased with increased storage time.

**Table 1:** Comparisons of apparent bovine plasma glucose concentrations from samples stored for different length of time and with different number of freeze-thaw cycles measured using both a peroxidase and glucose oxidase (PGO) and hexokinase (HK) assay^1^

Item	Mean	Mean difference^2^	BA 95% CI^3^	*t*-test 95% CI^4^	*P*-value^5^	r^6^
Time stored PGO						
ST2	2.91	—	—	—	—	—
ST3	2.82	0.08	−0.43, 0.59	0.04, 0.12	<0.01	0.83
ST4	2.89	0.02	−0.56, 0.61	−0.02, 0.07	0.32	0.82
ST16	2.74	0.17	−0.29, 0.62	0.13, 0.20	<0.01	0.87
Time stored HK						
ST2	2.34	—	—	—	—	—
ST3	2.16	0.16	−0.15, 0.47	0.13, 0.19	<0.01	0.97
ST4	2.53	−0.18	−0.51, 0.13	−0.21, −0.16	<0.01	0.98
ST16	2.25	0.08	−0.25, 0.42	0.05, 0.11	<0.01	0.97
Freeze-thawing PGO						
FTC1	2.92	—	—	—	—	—
FTC2	2.83	0.08	−0.40, 0.56	0.04, 0.12	<0.01	0.87
FTC3	2.97	−0.05	−0.69, 0.58	−0.10, −0.004	0.03	0.79
FTC4	2.82	0.10	−0.35, 0.55	0.07, 0.14	<0.01	0.89
Freeze-thawing HK						
FTC1	2.33	—	—	—	—	—
FTC2	2.18	0.15	−0.05, 0.34	0.13, 0.16	<0.01	0.99
FTC3	2.58	−0.26	−0.55, 0.03	−0.28, −0.24	<0.01	0.98
FTC4	2.30	0.02	−0.23, 0.27	0.002, 0.04	0.03	0.98

1 Storage length was either 2 (ST2), 3 (ST3), 4 (ST4) or 16 wk (ST16). Samples were either frozen and thawed 1 (FTC1), 2 (FTC2), 3 (FTC3), or 4 (FTC4) times. All mean and CI are presented as mmol/L.

2 Mean difference subtracted from ST2/FTC1.

3 The Bland–Altman (BA) CI of the mean difference.

4 The matched *t*-test 95% CI of the mean difference.

5 *P-*value for the matched *t*-tests.

6 Pearson correlation coefficient for each comparison to ST2/FTC1. All correlations had a *P*-value <0.01.

**Figure 1:**
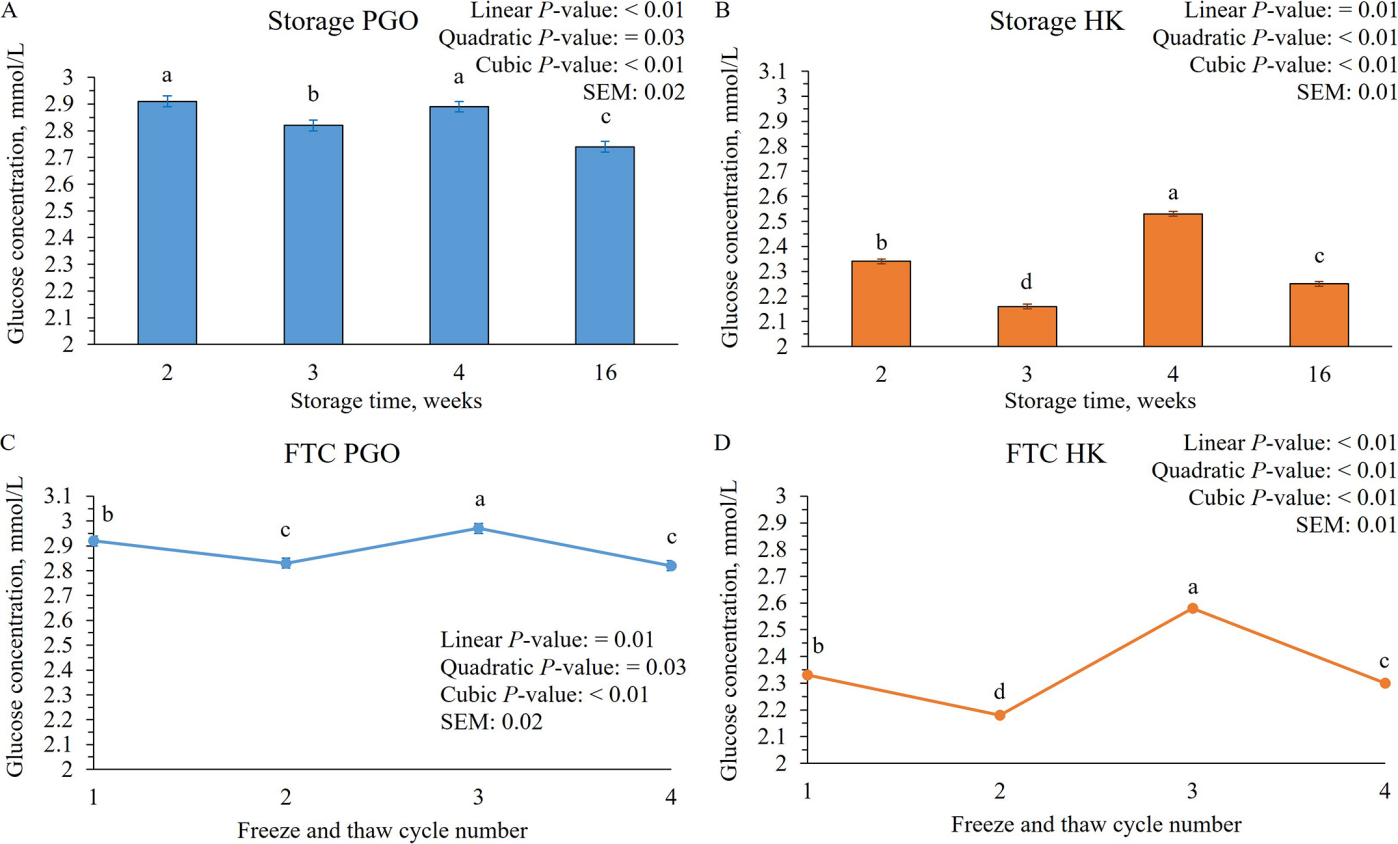
Comparisons of apparent bovine plasma glucose concentrations from samples undergoing increased storage time measured using both (A) peroxidase and glucose oxidase (PGO) and (B) hexokinase (HK) assays. Storage length was 2 (ST2), 3 (ST3), 4 (ST4), or 16 wk (ST16). Comparisons of apparent bovine plasma glucose concentrations from samples undergoing increasing freeze-thaw cycles (FTC) measured using both (C) PGO and (D) HK assays. Results are presented as LSM and SE, and different lowercase letters (a–d) represent differences in LSM of glucose across time points (*P* < 0.05).

The differences between FTC1 compared with FTC2, FTC3, and FTC4 with PGO were 0.08 (*P* < 0.01), −0.05 (*P* = 0.03), and 0.10 mmol/L (*P* < 0.01), respectively (Table 1; Figure 1). For samples analyzed with HK, the differences between FTC1 compared with FTC2, FTC3, and FTC4 were 0.15 (*P* < 0.01), −0.26 (*P* < 0.01), and 0.02 mmol/L (*P* = 0.03), respectively. Pearson correlations comparing FTC1 concentrations to FTC2, FTC3, and FTC4, had a significantly positive correlation and all had a correlation coefficient at or greater than 0.79 for PGO and 0.98 for HK assay. Likewise, the confidence intervals for the BA and the *t*-test mean differences of the PGO analysis for the freeze-thaw comparisons were wider in range compared with those of HK.

When comparing the assay systems used for analysis, we observed that the glucose concentrations differed throughout the weeks of analysis when comparing PGO and HK assays (Figure 2). On average, across the 4 time points considered, mean glucose concentration was greater (*P* < 0.01) in the PGO assay (2.86 mmol/L; 95% CI: 2.78, 2.93) than the HK assay (2.38 mmol/L; 95% CI: 2.31, 2.45).

**Figure 2:**
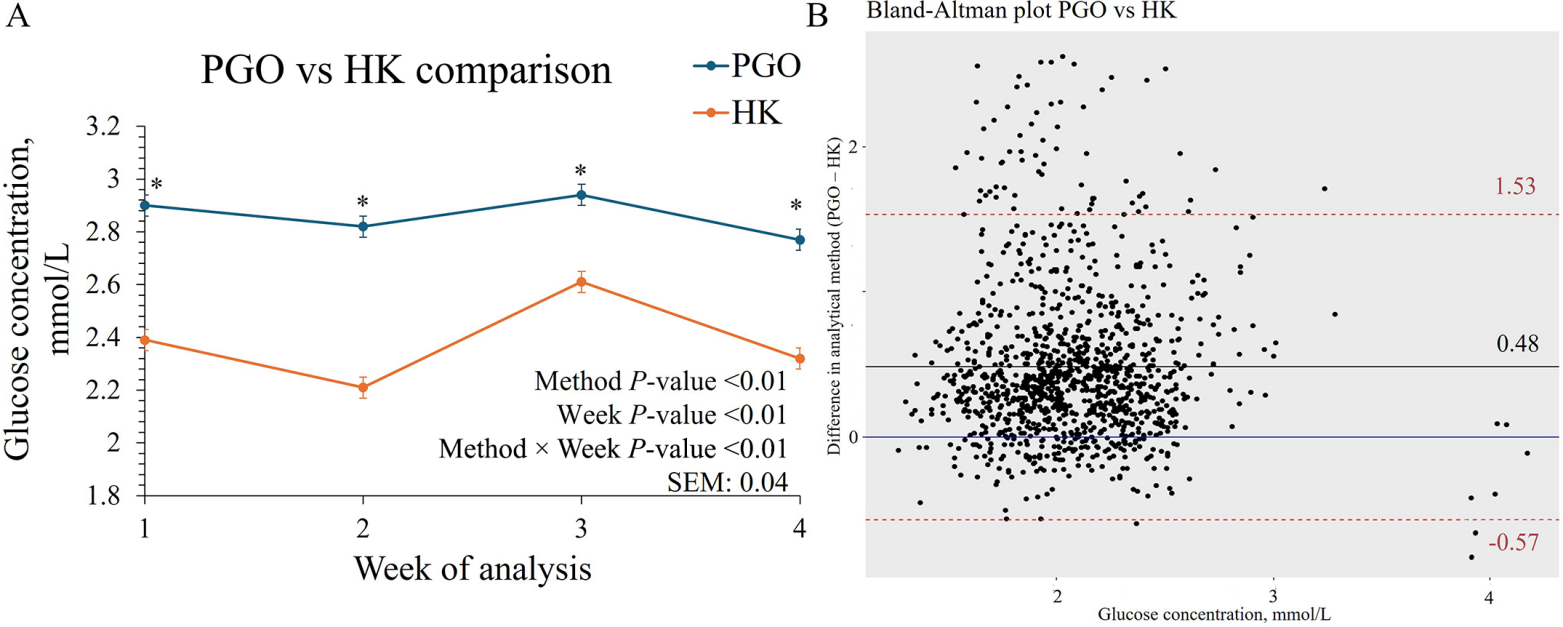
Comparison of 2 different assay systems for glucose concentration in plasma samples collected from cows. Panel A includes the mean concentration of the PGO assay and small-scale chemistry analyzer (HK) across the weeks of analysis and the standard error. Analysis time included all of the samples analyzed both for storage time and freeze-thaw cycles. *Indicates weekly comparisons between PGO and HK that were different based on Tukey adjusted pairwise comparisons (*P* < 0.05). Panel B includes the Bland–Altman plot for all of the samples measured using the PGO assay to the corresponding value obtained from the HK analysis.

When comparing glucose concentrations of the samples that had been frozen for different lengths of time, within the PGO assay, glucose concentrations decreased with increased storage time. The decrease in concentration was not a consistent gradual decrease; rather, it followed a cubic pattern of alternating decreases and increases of minimal concentration differences. The concentrations quantified at 3 wk, 4 wk, and 16 wk of storage were 0.08, 0.02, and 0.17 mmol/L, respectively, less than the concentration quantified after 2 wk of storage. Considering the inconsistent decreases in concentration, it is unlikely that increasing storage time up to 16 wk causes a biologically relevant change in the glucose quantification of samples collected with NaF. Additionally, 16 wk is a relatively short period compared with longer research trials and may have not been enough to detect potential changes in glucose concentration.

For the HK assay, the glucose concentration after 3 wk of storage was 0.16 mmol/L lower than after 2 wk, but at 4 wk, it was 0.18 mmol/L higher. After 16 wk, the concentration was 0.08 mmol/L lower than at 2 wk. The increased concentration quantified after 4 wk of storage is the opposite of what we had hypothesized. However, Haid et al. (2018) observed that in human plasma samples stored at −80°C hexose concentration quantified over 5 yr increased by an average of 7.9%. The authors suggested that the increase in hexose could be due to the release of sugar moieties from lipoprotein particles such as low-density lipoprotein, high-density lipoprotein, and very-low-density lipoprotein. This might be an explanation for the observed increase in glucose concentration quantified over time with HK; however, given that the increase observed in the current study only occurred at one time point after the first quantification, random error or assay variation may be a more viable explanation for this observation. Similarly to what was previously mentioned with PGO, these fluctuating differences may be indicative that storage time up to 16 wk does not have a biologically relevant impact on glucose quantification.

The plasma glucose concentration of dairy cows has been reported to range from 1.89 to 6.05 mmol/L with a mean of 3.41 mmol/L (Lopes et al., 2019). The HK assay calibrates between 4.38 to 6.32 mmol/L and 14.76 to 18.54 mmol/L using a 2-point curve, likely missing cows' glucose levels. To standardize comparisons, PGO assay standards were used for both assays in this study. The greatest agreement between the PGO and HK concentrations occurred at the third time that samples were analyzed, and the difference between assays was 0.33 mmol/L.

Although comparison of freeze-thaw cycles resulted in different values between the 2 assays, they followed a similar pattern to that of storage time. It was observed that across analysis times, the concentrations quantified for the same aliquot, which had been previously thawed, yielded both lower and higher glucose concentrations. Overall, for the freeze-thaw comparison the greatest difference observed for freeze-thaw cycles for the PGO assay was between FTC3 and FTC4 with a 0.15 mmol/L change in the concentrations quantified. The current study was powered to detect a small difference that could potentially be considered biologically irrelevant (Mair et al., 2016). The statistical difference in the current study may indicate that in other studies, freeze-thaw cycles during sample analysis may add variation to quantification of glucose.

For the freeze-thaw comparison for HK, the largest difference observed was between FTC2 and FTC3. The difference was a 0.40 mmol/L increase in the concentration, and this would be both statistically different and biologically relevant. In contrast to our results, a previous study considering isolated effects of freezing and thawing human plasma on measured concentrations of 87 metabolites observed that the metabolites are quite stable when freezing and thawing occurred rapidly (Buchanan et al., 2022). Our research study suggests that caution to minimize freeze-thaw cycles of samples should be taken when measuring glucose concentration in bovine blood, and when possible, samples undergoing the same analysis should undergo the same number of freeze-thaw cycles.

The current study demonstrated that variation in sample concentrations obtained from the HK was less than that of the PGO assay, as there were larger CI with PGO. This is likely because the HK assay used semi-automated chemistry analyzer with automated pipetting of samples unit that measured glucose concentrations compared with the PGO assay which used manual pipetting and was therefore subject to greater human error in sample and reagent volumes. In a study comparing a 96-well plate adapted analysis and a semi-automated chemistry analyzer with a gold standard method using all the same reagents for nonesterified fatty acid quantification (Abuelo et al., 2020), the CI for the chemistry analyzer were smaller than those of the plate assay. Thus, if all samples can be analyzed within the time of a single calibration, it is likely that the HK would be a more optimal assay system, as it can be more precise within each calibration because the automated system is less susceptible to pipetting error. However, if there is interest in comparing glucose concentrations measured across a greater period, it is likely that the PGO assay would a preferred assay because the glucose concentrations measured by this assay were more consistent over a longer period of time.

Some limitations of the study include that the samples were all stored at −20°C; thus we cannot assess how our results would compare with storage at different temperatures. Additionally, the results observed in the current study may not be generalizable to other species, especially for the HK assay, due to different glucose concentrations among different species. Finally, there are several factors that may lead to storage of samples for longer than 16 wk, including collection of blood samples from the dry period throughout the completion of lactation, running a study across 2 calving years, or unforeseen delays in the ability to analyze samples after collection. Future studies could consider the influence of longer storage times than the one used in the current study.

Overall, storage time and freeze-thaw cycles of up to 16 wk have minimal influence on the concentration of glucose in samples collected with vials containing NaF as the anticoagulant. Additionally, the concentrations of glucose measured varied depending on the assay system used. The PGO assay was a more consistent assay system over time, whereas the HK had more glucose concentration variation over time. The HK assay was more precise for measuring glucose concentrations within each time point compared with the PGO assay. Thus, if all samples will be measured in a short time frame HK offers the advantage of improved precision because of automation, the PGO assay is a better alternative for samples analyzed at different time points, especially if all the standards are created at one time and stored for future analysis. The assays are not comparable, and it is likely they should not be compared across studies. Future studies should consider the influence of the use of the same enzymatic protocol with different analysis methods such as the plate or a small-scale chemistry analyzer.
